# Recurrence of IgA nephropathy in a kidney transplant patient successfully treated with iptacopan—a case report

**DOI:** 10.3389/fimmu.2026.1803607

**Published:** 2026-05-01

**Authors:** Jay Pandav, Basheer Kummangal, Justin Smith, Angelo De Mattos, Megan Stack, Zoha Ahmad, Nicole Andeen, Vanderlene Kung, Ali Olyaei, Shehzad Rehman

**Affiliations:** Department of Medicine, Division of Nephrology and Hypertension, Oregon Health and Science University, Portland, OR, United States

**Keywords:** IgA nephropathy, iptacopan, kidney biopsy, kidney transplant, recurrence

## Abstract

IgA nephropathy recurrence after kidney transplantation is a common complication, which is most often detected histologically. Recurrence after transplant affects long-term graft survival, with prognosis being worse with increasing levels of proteinuria, a higher histological activity, and concurrent rejection. Treatment in kidney transplant patients is similar to that in native kidneys: using renin–angiotensin–aldosterone system (RAAS) blockade agents, steroids, and increased immunosuppression. Iptacopan, an inhibitor of alternative complement pathway factor B, has demonstrated reduction in proteinuria and complement activity in native IgAN. However, it has not been studied in recurrent IgAN after kidney transplant. We present a case of recurrent IgAN refractory to conventional therapy with clinical and histological improvement after treatment with iptacopan. A 39-year-old male patient underwent a deceased donor kidney transplantation. A 3-month biopsy showed features of recurrent IgAN in the transplanted kidney. Following treatment for rejection, subsequent biopsies demonstrated active IgAN with worsening proteinuria, which was treated with rituximab, cyclophosphamide, and steroids. Despite these interventions, there was no improvement in IgAN or proteinuria. At 40 months post-transplant, the patient was started on iptacopan, after which a follow-up biopsy showed resolution of active IgA activity. The patient has tolerated iptacopan well and has not had any adverse events or serious infections.

## Introduction

IgA nephropathy recurrence after kidney transplantation is a common complication, with rates varying between 20% and 60% depending on the biopsy practices and duration of follow-up (1). Many of these are clinically silent, with only histological recurrence diagnosed in surveillance biopsies (1, 2). Male sex, younger recipient age, rapid progression of native IgAN, the presence of crescents on native kidney biopsy, living related donation, and rapid steroid taper post-transplantation are some of the factors known to increase the risk of IgAN recurrence (1, 3–5). It adversely affects long-term graft survival, with prognosis being worse with increasing levels of proteinuria, a higher histological activity, and concurrent acute rejection (1, 2, 6, 7). Treatment is similar to that of native IgAN, with the use of renin–angiotensin–aldosterone system (RAAS) blockade and intensifying steroids as cornerstones. Some studies have also described the use of sodium–glucose co-transporter (SGLT2) inhibitors, cyclophosphamide, and increasing immunosuppression (1, 2, 6, 8). Complement activation plays a key role in the mechanism of glomerular injury in IgAN and thus has been a recent focus of therapeutic investigations (9, 10). Iptacopan is a novel inhibitor of factor B in the alternative complement pathway. Recent phase 2 and 3 trials of iptacopan have demonstrated reduction in proteinuria and markers of complement activation in native IgAN with a good safety profile (11, 12). However, it has not been thoroughly investigated in the treatment of post-transplant recurrence of IgAN. Here, we describe a case of recurrent IgAN after kidney transplantation refractory to conventional therapy, with clinical and histological improvement after treatment with iptacopan.

## Case description

**Figure 1:**
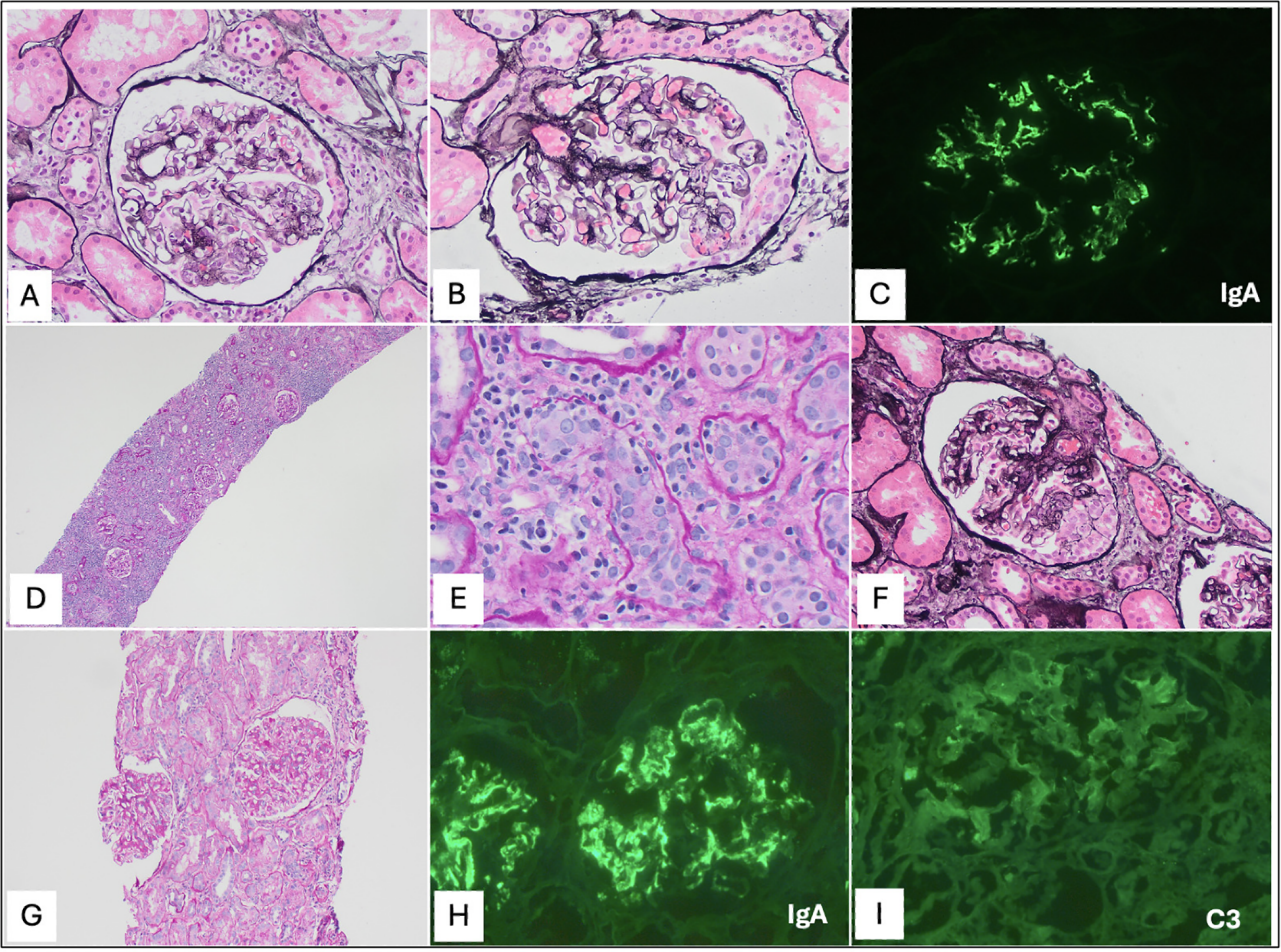
Histopathology. 12-month biopsy: **(A)** Segmental endocapillary and **(B)** focal crescent formation - Jones silver 200x. **(C)** Immunofluorescence showing bright granular mesangial staining for IgA. 17-month biopsy: **(D)** Worsening Banff IB T-cell mediated rejection (TCMR) with diffuse tubulointerstitial inflammation - PAS 100x, **(E)** severe tubulitis - PAS 400x. 33-month biopsy: **(F)** Histologic resolution of TCMR but re-development of active IgAN, which became more severe in activity (up to 24% crescents) and chronicity due to IgAN. 48-month biopsy: **(G)** After iptacopan treatment, biopsy showed mesangial proliferation (PAS 200x) and segmental sclerosis but no acute inflammatory lesions of IgAN such as endocapillary hypercellularity, necrosis or crescents. **(H)** Immunofluorescence showing persistent strong granular mesangial and segmental peripheral capillary wall staining for IgA. **(I)** C3 immunofluorescence showed trace staining in glomeruli post-iptacopan, unchanged from prior biopsies showing trace or zero staining for C3.

**Figure 2:**
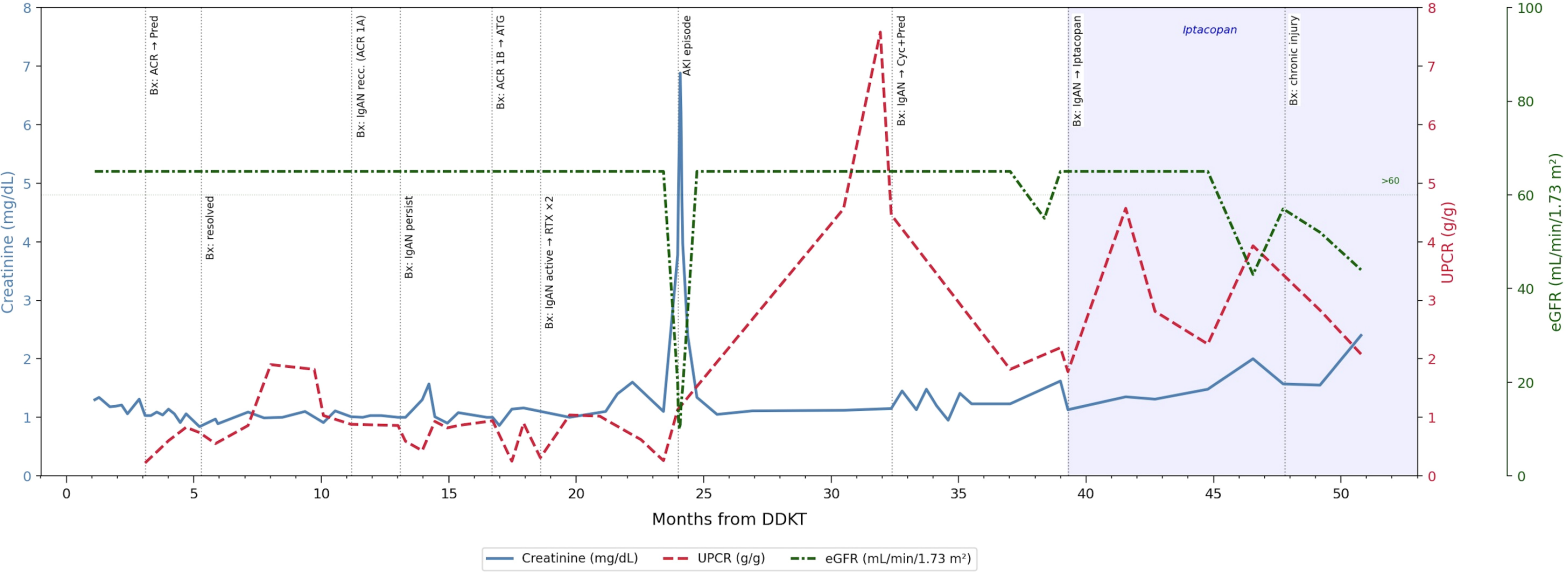
A pictural timeline of the patient’s graft function [creatinine (Cr), estimated glomerular filtration rate (eGFR), and proteinuria] observed over time. The *X*-axis represents the time from transplant in months. The *Y*-axis represents the laboratory values.

A 39-year-old male patient with a history of kidney disease due to biopsy-proven crescentic IgAN underwent a deceased donor kidney transplantation. He underwent induction with anti-thymocyte globulin (total dose, 4.5 mg/kg) and was on maintenance immunosuppression with tacrolimus, mycophenolate mofetil, and prednisone. His early postoperative course was unremarkable, and he had excellent graft function [nadir creatinine (Cr) = 0.8 mg/dl, no proteinuria or hematuria]. At 11 months post-transplant, he had an asymptomatic Epstein–Barr virus (EBV) infection (peak whole blood viral load = 47,300 copies/ml), for which the mycophenolate dose was reduced and he was treated with one dose of rituximab, with good results. The infection resolved; however, at 12 months post-transplant, he underwent a for-cause kidney biopsy [Cr = 1.0 mg/dl, urine protein/creatinine concentration (UPCR) = 1.03 g, (+) hematuria], which showed Banff grade 1A T-cell-mediated rejection (TCMR) with features of IgA recurrence (Oxford classification M0 E1 S1 T0 C1), with immunofluorescence showing 4+ granular mesangial IgA staining (**Figures 1A–C**). The patient was treated with oral prednisone 60 mg daily for 5 days and was maintained on 10 mg daily. At 17 months post-transplant, a follow-up biopsy showed Banff grade 1B TCMR with no light microscopic evidence of IgAN, but with immunofluorescence showing 2+ granular IgA mesangial staining (**Figures 1D, E**). TCMR was treated with anti-thymocyte globulin. At 19 months post-transplant, a follow-up biopsy showed active IgAN (Oxford classification M1 E1 S0 T0 C1, with immunofluorescence showing granular 4+ global mesangial and segmental IgA staining). He was treated with two doses of rituximab. At 33 months post-transplant, he was found to have worsening proteinuria at 4.6 gm/gm. A kidney biopsy showed increased IgA activity (Oxford classification M1 E1 S1 T0 C1, active cellular crescents in 24% of glomeruli and segmental scars in 24% glomeruli). There was also an interval increase in interstitial fibrosis and tubular atrophy at 20% compared with 10% on the previous biopsy (**Figure 1F**). The patient received intravenous cyclophosphamide for 6 weeks and an oral prednisone pulse (80 mg daily, followed by gradual taper over 6 months to a maintenance of 10 mg daily). At 40 months post-transplant, a biopsy showed IgAN with features of reduced activity and increased chronicity—fibrocellular crescents in 15% of glomeruli and 40% glomeruli showing fibrous scars/segmental sclerosis. Immunofluorescence showed 3–4+ granular mesangial IgA staining. Based on these findings, the patient was started on iptacopan 200 mg daily after receiving appropriate vaccination (for encapsulated organisms such as meningococcus and pneumococcus) and starting penicillin prophylaxis. A follow-up biopsy was performed at 48 months post-transplant, which showed features of chronic injury, but resolution of active IgA inflammatory activity (Oxford classification M1 E0 S1 T1 C0), with immunofluorescence showing 3–4+ mesangial IgA staining similar to the previous biopsy (**Figures 1G–I**). To date, the patient is still on iptacopan therapy, which will be continued for another 6 months, after which a decision to continue or withdraw treatment will be made based on repeat biopsy findings and laboratory parameters. Thus far, the patient has tolerated iptacopan well and has not experienced any serious infections or adverse reactions. His kidney function has remained stable, and there has been some improvement in proteinuria as well. **Figure 2** shows a timeline of the patient’s serum Cr, estimated glomerular filtration rate (eGFR), and UPCR since the time of transplantation.

## Discussion

This is our institution’s first experience with iptacopan demonstrating both histologic and clinical improvement in post-transplant recurrent IgAN refractory to conventional therapy. Our patient had active disease that persisted despite treatment with rituximab, cyclophosphamide, and corticosteroids. However, following initiation of iptacopan, there was resolution of active glomerular inflammation. Moreover, stabilization of proteinuria was also accomplished, suggesting a favorable therapeutic clinical effect. While chronic histological changes persist (and are irreversible), the attenuation of active inflammatory activity supports a potential disease-modifying role for factor B inhibition. Our findings align with the evolving understanding of complement activation as a central driver of IgAN and its recurrence post-transplantation. We did an extensive literature review to determine whether other centers have had any experience with the use of iptacopan for the treatment of IgAN recurrence in kidney transplant. To date, there is very limited literature, and we only found two papers that demonstrated similar experience. Al Jurdi et al. published a case series of three patients who had recurrence of IgAN in their transplanted kidneys (13). All patients received iptacopan for more than 3 months, after which two patients demonstrated significant reductions in proteinuria and resolution of microscopic hematuria. One individual developed progressive graft dysfunction, likely due to a more aggressive or atypical disease phenotype. However, the duration of follow-up for all patients was relatively short (4 months). Sanna et al. described a case series of five patients who experienced recurrence of IgAN in their kidney allograft (14). Three of the five patients were treated with iptacopan only, whereas the other two patients were treated with iptacopan and steroid pulse or steroid pulse and plasmapheresis. Within 6–12 months, iptacopan led to a marked reduction in terminal complement and C3 deposition in the kidney allografts. Complement inhibition may represent a promising therapy in cases unresponsive to standard treatments. Future prospective studies and controlled trials are warranted to establish the safety, efficacy, optimal treatment duration, and long-term graft outcomes associated with this therapeutic approach.

## Data Availability

The original contributions presented in the study are included in the article/supplementary material. Further inquiries can be directed to the corresponding author.

## References

[1] Jäger C Stampf S Molyneux K Barratt J Golshayan D Hadaya K . Recurrence of IgA nephropathy after kidney transplantation: experience from the Swiss transplant cohort study. BMC Nephrol. (2022) 23. doi: 10.1186/s12882-022-02802-x. PMID: 35538438 PMC9088042

[2] Uffing A Pérez-Saéz MJ Jouve T Bugnazet M Malvezzi P Muhsin SA . Recurrence of IgA nephropathy after kidney transplantation in adults. Clin J Am Soc Nephrol. (2021) 16:1247–55. doi: 10.2215/CJN.00910121. PMID: 34362788 PMC8455056

[3] Li Y Tang Y Lin T Song T . Risk factors and outcomes of IgA nephropathy recurrence after kidney transplantation: a systematic review and meta-analysis. Front Immunol. (2023) 14:1277017. doi: 10.3389/fimmu.2023.1277017. PMID: 38090563 PMC10713786

[4] Clayton P McDonald S Chadban S . Steroids and recurrent IgA nephropathy after kidney transplantation. Am J Transplant. (2011) 11:1645–9. doi: 10.1111/j.1600-6143.2011.03667.x. PMID: 21797974

[5] Avasare RS Rosenstiel PE Zaky ZS Tsapepas DS Appel GB Markowitz GS . Predicting post-transplant recurrence of IgA nephropathy: the importance of crescents. Am J Nephrol. (2017) 45:99–106. doi: 10.1159/000453081. PMID: 28056461 PMC5296401

[6] Moroni G Longhi S Quaglini S Gallelli B Banfi G Montagnino G . The long-term outcome of renal transplantation of IgA nephropathy and the impact of recurrence on graft survival. Nephrol Dial Transplant. (2013) 28:1305–14. doi: 10.1093/ndt/gfs472. PMID: 23229925

[7] Kavanagh CR Zanoni F Leal R Jain NG Stack MN Vasilescu ER . Clinical predictors and prognosis of recurrent IgA nephropathy in the kidney allograft. Glomerul Dis. (2022) 2:42–53. doi: 10.1159/000519834. PMID: 35450416 PMC9017582

[8] Kwon H Son SH Kong JM . SGLT2 inhibitors reduce the rate of decline in the estimated glomerular filtration rate of kidney transplant patients with recurrent or De Novo glomerulonephritis: TH-PO742. J Am Soc Nephrol. (2024) 35:10.1681/ASN.202425vyres0. doi: 10.1681/ASN.202425vyres0. PMID: 31941820

[9] Medjeral-Thomas NR Cook HT Pickering MC . Complement activation Medjeral-Thomas NR, Cook HT, Pickering MC. Complement activation in IgA nephropathy. Semin Immunopathol. (2021) 43:679–90. doi: 10.1007/s00281-021-00882-9. PMID: 34379175 PMC8551128

[10] Schubart A Anderson K Mainolfi N Sellner H Ehara T Adams T . Small-molecule factor B inhibitor for the treatment of complement-mediated diseases. Proc Natl Acad Sci USA. (2019) 116:7926–31. doi: 10.1073/pnas.1820892116. PMID: 30926668 PMC6475383

[11] Zhang H Rizk DV Perkovic V Maes B Kashihara N Rovin B . Results of a randomized double-blind placebo-controlled Phase 2 study propose iptacopan as an alternative complement pathway inhibitor for IgA nephropathy. Kidney Int. (2024) 105:189–99. doi: 10.1016/j.kint.2023.09.027. PMID: 37914086

[12] Perkovic V Barratt J Rovin B Kashihara N Maes B Zhang H . Alternative complement pathway inhibition with iptacopan in IgA nephropathy. N Engl J Med. (2025) 392:531–43. doi: 10.1056/NEJMoa2410316. PMID: 39453772

[13] Al Jurdi A Cohen Bucay A Nissaisorakarn P Gilligan H Avillach CT Klepeis M . Early experience with iptacopan for recurrent IgA nephropathy after kidney transplantation. Kidney Med. (2025) 8:101189. doi: 10.1016/j.xkme.2025.101189. PMID: 41608295 PMC12835414

[14] Sanna E Nicassio SR Mella A Mingozzi S Manzione AM Dolla C . Targeting the alternative complement pathway by iptacopan abrogates C3 and strongly reduces C5b-9 deposition within glomeruli in IgA nephropathy recurrence after kidney transplantation. Am J Transplant. (2025) 25:2651–7. doi: 10.1016/j.ajt.2025.08.015. PMID: 40854489

